# Paeonin extracted from potatoes protects gastric epithelial cells from H_2_O_2_-induced oxidative damage *in vitro* by PI3K/Akt-mediated Nrf2 signaling pathway

**DOI:** 10.1038/s41598-018-28772-5

**Published:** 2018-07-18

**Authors:** Jiping Xiao, Bo Chen, Qiong Wang, Lijuan Yang, Huachun Guo

**Affiliations:** 1 College of Agronomy and Biotechnology, Yunnan Agricultural University, Kunming, Yunnan 650201 China; 2 Experiment Center for Medical Science Research, Kunming Medical University, Kunming, Yunnan 650500 China; 3 College of Mechanical & Electrical Engineering, Yunnan Agricultural University, Kunming, Yunnan 650201 China; 4 Department of Pathology and Pathophysiology, Kunming Medical University, Kunming, Yunnan 650500 China

## Abstract

In this study, it is aimed to investigate the antioxidant mechanism of new extracts from potatoes. Four pigments, namely, Petunin, Paeonin, Malvidin and Pelargonidin, were extracted from potatoes by high performance liquid chromatography (HPLC). Our results showed that the cellular morphology and cell viability were significantly altered in gastric mucosal epithelial cells (GES-1) treated with different hydrogen peroxide (H_2_O_2_) concentrations over time (*P* < 0.05). Paeonin presented the strongest anti-oxidative effects on H_2_O_2_-treated cells, in both a dose- and time-dependent manner, determined by ARE-luciferase activity and HO-1 mRNA expression. After pre-treatment with Paeonin in H_2_O_2_-exposed cells, Keap1, Nrf2, HO-1 and NQO1 protein expressions were remarkably up-regulated. Furthermore, immunostaining of Nrf2 expression was obviously elevated in the H_2_O_2_ + Paeonin group over time. The GSH content in the H_2_O_2_ + Paeonin group was notably lower than that in the H_2_O_2_ + Paeonin + GSK690693 group. Paeonin promoted cell cycle with augmented Cyclin D1 and p27 protein expressions. Moreover, Paeonin suppressed apoptosis with increased Bcl2, total Caspase3 and total Caspase8 protein expressions and decreased Bax, p-Caspase3 and p-Caspase8 protein expression in H_2_O_2_-treated cells. These results suggested that Paeonin might exert an anti-oxidative role by activating Nrf2 signaling pathway with the changes of cell cycle and apoptosis.

## Introduction

Gastric diseases, including chronic gastritis, adenocarcinoma, duodenal and gastric ulceration and gastric mucosa-associated lymphoid tissue lymphoma, pose great financial burden on the healthcare system and significantly diminish quality of life^1,2^. Thus, international attention is needed to help address the worldwide burden of disease from these illnesses. A growing body of experimental and clinical evidence suggests that in most cases, chronic increased oxidative stress is the primary gastric pathologic feature that plays an important role in the multiple progressions of gastric diseases^3,4^. For example, ethanol-induced gastric ulcer in rats could cause elevated lipid peroxidation and malonic dialdehyde, which ultimately resulted in mucosal oxidative stress impairment; however, after administration of anti-oxidants and/or anti-oxidant enzymes, there was protection against ethanol-induced gastric ulcer via inhibition of oxidative stress damage^5^.

Oxidative stress is defined as the imbalance between reactive oxygen species (ROS) generation and the endogenous anti-oxidant defense system. Under normal physiological conditions, ROS produced by mammalian cells can be degraded effectively by anti-oxidant defense molecules, such as glutathione, superoxide dismutase, catalase, peroxidase, and thioredoxin protein^6,7^. Nevertheless, an aberrant and excessive generation of ROS may result in serious oxidative damage to indispensable biomolecules, including proteins, lipids, and nucleic acids^8,9^. Among various ROS, hydrogen peroxide (H_2_O_2_) is a stable, small and uncharged molecule, which acts as a physiological second messenger and freely diffuses across cell membranes^9,10^. Moreover, H_2_O_2_ produces at nearly every stage of the oxidative cycle and initiated lipid peroxidation, mitochondria injury, and DNA damage^11^, and is also widely applied to induce oxidative stress *in vitro*^12^.

According to the above-described studies, if intervention strategies can ameliorate the processes of oxidative stress or its related pathway, they may be applicable for improving clinical symptoms of gastric diseases^13,14^. However, nuclear factor-E2-related factor-2 (Nrf-2)/anti-oxidant response element (ARE) signaling pathway, which could control the expression of genes whose protein products are involved in the detoxification and elimination of reactive oxidants, is a major mechanism in the cellular defense against oxidative stress^15^. Additionally, many researchers stated that antioxidant-rich foods, such as apple^16^, strawberry^17^, tomato, carrot, etc., are able to scavenge ROS and eventually reduce the risk for gastric mucosal oxidative injury. Hence, in the current study, we attempted to explore the anti-oxidative roles of the ingredients or extracts from potatoes that can suppress oxidative stress damages, and to further investigate the underlying mechanisms implicated in Nrf2/ARE signaling pathway of these materials in inhibiting oxidative stress injuries.

## Materials and Methods

### Cell culture and hydrogen peroxide (H_2_O_2_) treatments

Human gastric mucosal epithelial cell line, GES-1 cells, purchased from American Type Culture Collection (ATCC), were cultivated in Roswell Park Memorial Institute 1640 (RPMI1640; Gibco, Germany) complete media, supplemented with 10% fetal bovine serum (FBS; Gibco, Germany), 1% penicillin/streptomycin, 4 mM L-glutamine, and 4500 mg/l glucose at 37 °C in a humidified atmosphere with 5% CO_2_. The media were changed every 2 days and when GES-1 cells reached 80~90% confluence, 0.25% trypsin (Sigma, USA) was used to digest cells for the subculture.

GES-1 cells were seeded into 6-well plates at a density of 1 × 10^5^ cells/well and placed under a 5% CO_2_ humidified incubator at 37 °C overnight. On reaching 80% confluence, GES-1 cells were treated with 0 μM, 100 μM, 200 μM and 400 μM H_2_O_2_, for 24 h. Subsequently, according to the examination of cell viability, we chose an appropriate concentration for the following experiments.

### Cell viability assay

Briefly, 3-(4,5-dimethylthiazol-2-yl)-2,5-diphenyltetrazolium bromide (MTT) assay was conducted to determine the cell viability of GES-1 cells treated with different concentrations of H_2_O_2_ using an MTT kit (Promega, USA) in accordance with the manufacturer’s instructions. Then, the cultured GES-1 cells with different concentrations of H_2_O_2_ were plated in 96-well plates for 24 h and then incubated with 20 μl of 5 mg/ml MTT reagent at 37 °C for 4 h. Afterwards, 150 μl of dimethyl sulfoxide (DMSO) solution was added to every well to dissolve the purple formazan crystals, accompanied by continuous shaking at room temperature for 15 min. Finally, the absorbance of each well, including the blank well without cells, was measured at a wavelength of 490 nm by a spectrophotometer (Bio-Rad, USA). Moreover, according to the results of the above experiment, we selected the minimum of cell viability in GES-1 cells with H_2_O_2_ treatments, and further examined the cell viability in GES-1 cells incubated with the chosen H_2_O_2_ concentration for 0 h, 3 h, 6 h, 12 h and 24 h.

### Extraction of pigments from the potatoes was analyzed by High Performance Liquid Chromato-graphy (HPLC)

The pigments were extracted from potatoes by using chemical reagents purchased from Fisher Scientific, USA according to the related manufacturer’s protocols. Then, the extracted pigments were analyzed on an Agilent HPLC instrument (Agilent, USA), which consisted of a G1311A Quaternary Pump, a G1322A degasser, a G1314A Ultraviolet (UV) Detector, a G1316A column, and the HPLC ChemStation software. Analysis condition was as follows: a column C_18_, 2.1 × 7.5 mm and a guard column (4.6 mm internal diameter) (Agilent, USA) were performed; the mobile phase was A solution (water acidified with formic acid, pH = 3.0) and B solution (methanol) in a step gradient manner with a flow rate of 0.5 ml/min; detection wavelength was observed from 525 nm.

### Anti-oxidant response element (ARE)-luciferase activity measurement

GES-1 cells were seeded into 24-well plates and ARE-luciferase reporter plasmid obtained from Biolab, China, was transfected into GES-1 cells using Lipofectamine^TM^ 2000 (Invitrogen, USA) following the manufacturer’s recommendations. After 24 h of transfection, the transfected cells were treated with 400 μM H_2_O_2_ alone and 400 μM H_2_O_2_ + four extracted pigments, respectively, for 24 h. Eventually, the activity of ARE-luciferase was measured by adding Luciferase Assay Reagent (Promega, USA) according to the manufacturer’s guidelines at a wavelength of 490 nm by a spectrophotometer. Furthermore, according to the results of the above experiment, we selected the maximum of ARE-luciferase activity in GES-1 cells with H_2_O_2_ + pigments treatment, and further examined the ARE-luciferase activity in GES-1 cells incubated with different concentrations of chosen pigments for 1 h, 2 h, 4 h, 8 h, 12 h and 24 h.

### Quantitative reverse transcription-polymerase chain reaction (qRT-PCR) detection

Total RNA of the harvested GES-1 cells with different treatments as above described were isolated with Trizol Reagent (TIANGEN, China), in accordance with the manufacturer’s instructions, and the RNA preparations were cleared of contaminating genomic DNA by DNase treatment (Thermo, USA). Single-strand complementary DNA (cDNA) was synthesized from 0.5 μg of total RNA using a PrimeScript RT reagent kit (Takara, Japan). Quantitative analysis of heme oxygenase-1 (HO-1) mRNA was performed on a 7500 Fast Real-time PCR system (Applied Biosystems, USA) using a SYBR^®^ Premix Ex Taq™ II (Tli RNaseH Plus) kit according to the manufacturer’s protocols. The PCR conditions consisted of 1 cycle of denaturing at 95 °C for 30 s, then 40 cycles of denaturing at 95 °C for 5 s, annealing at 60 °C for 34 s (with data collection at the end of 60 °C step at each cycle), and dissociation at 95 °C for 15 s, 60 °C for 1 min and 95 °C for 15 s. HO-1 quantitative data were normalized to the expression level of 18S rRNA and calculated with the 2^−ΔΔCt^ method^18^. The sequences of primers used for qRT-PCR were as follows: HO-1 forward primer, 5′-TGAAGGAGGCCACCAAGGAGGA-3′, HO-1 reverse primer, 5′-AGAGGTCACCCAGGTAGCGGG-3′; 18S rRNA forward primer, 5′-CCTGGATACCGCAGCTAGGA-3′, 18 S rRNA reverse primer, 5′-GCGGCGCAATACGAATGCCCC-3′.

### Western blotting (WB)

The samples of GES-1 cells incubated with 400 μM H_2_O_2_ + 200 μg/ml Paeonin for 0 h, 1 h, 2 h, 4 h, 8 h, 12 h and 24 h were collected for measurements of cell viability by MTT assay, which was described above, and Nrf signaling pathway-related proteins by WB assay. Whole-cell lysate from all samples was prepared in pre-cooled radioimmunoprecipitation assay (RIPA) lysis buffer (Thermo, USA) containing 50 mM Tris-HCl (pH 7.5), 150 mM NaCl, 0.1% Nonidet P-40, and a mixture of protease inhibitors or phosphatase inhibitor on ice for 40 min. The total protein concentration was quantified with a BCA protein assay kit (Beyotime, China), using bovine serum albumin (BSA) as a standard. The cell lysates were mixed with 2× loading buffer (Beyotime, China), denatured by boiling for 8 min and then resolved by 8~10% sodium dodecyl sulfate-polyacrylamide gels for electrophoresis (SDS-PAGE) (30 μg/well). After running for 1.5 h at a constant voltage of 120 V, the proteins were transferred onto polyvinylidene fluoride (PVDF) membranes for 2 h at a constant current of 200 mA, blocked in 5% non-fat milk for 2 h at room temperature, and incubated with rabbit anti-Nrf2 primary antibody (1:1000 dilution; Abcam, USA), rabbit anti-HO-1 primary antibody (1:1000 dilution; Abcam, USA), rabbit anti-NQO1 primary antibody (1:2000 dilution; Abcam, USA), rabbit anti-phospho-Akt (p-Akt) primary antibody (1:1000 dilution; Abcam, USA), rabbit anti-Akt primary antibody (1:2000 dilution; Abcam, USA), or mouse anti-β-actin (1:1000 dilution, as an internal control; Abcam, USA) overnight at 4 °C. After extensive washing with Tris-buffered saline with 0.1% Tween-20 (TBST) three times, the membranes were probed with the corresponding secondary antibodies, including goat anti-mouse IgG and goat anti-rabbit IgG (both 1:12000 diluted in 5% non-fat milk; Abmart, USA), for 1 h at room temperature, and then washed again with TBST three times. Finally, the blots were detected by enhanced chemiluminescence reagents (Beyotime, China) by an ImageQuant LAS 4000 mini system (GE Healthcare, Japan), and the densitometry of protein bands was analyzed by ImageJ software.

### Glutathione (GSH) content examination

GES-1 cells were plated onto 6-well dishes (1 × 10^5^ cells/well), and the next day cells were exposed to H_2_O_2_ alone, H_2_O_2_ + Paeonin and H_2_O_2_ + Paeonin + GSK690693 (an inhibitor of PI3K/Akt signaling pathway) for 24 h. Then, scraped cells were lysed in 5% metaphosphoric acid and sonicated on ice. After being centrifuged at 12000rmp for 10 min, the intracellular GSH levels in the supernatants were measured by using a commercial GSH determination kit (Nanjing Jiancheng Biotechnology, China) following the kit manuals. Absorbances of each sample were read at 400 nm using a spectrophotometer.

### Immunofluorescence (IF) microscopy

GES-1 cells were seeded on 12-well plates with pre-placed sterile glass coverslips and left to attach overnight in a 5% CO_2_ incubator at 37 °C. The next day, cells were incubated with H_2_O_2_ alone and H_2_O_2_ + Paeonin for 2 h, 8 h and 12 h. After treatments at the indicated time, cells were washed with phosphate buffered saline (PBS) twice, fixed with 4% paraformaldehyde for 30 min at room temperature, and then permeabilized with 1% Triton X-100 for 10 min. After blocking with 5% BSA for 30 min at 37 °C, the cells were stained with the anti-Nerf primary antibody (1:100 dilution) overnight at 4 °C. The cells were washed with PBS three times followed by PE-conjugated goat anti-rabbit secondary antibody (1:1000 dilution; Abcam, USA) incubation for 1 h at 37 °C. After 3 more washes with PBS, cells were counterstained with 1 mg/ml of DAPI (Beyotime, China) for 5 min and the coverslips were mounted with antifade reagent (Solarbio, China). Ultimately, the images were observed and captured by a laser-scanning confocal microscope (Leica, Germany).

### Flow Cytometry

To detect cell cycle and apoptosis of GES-1 cells treated with H_2_O_2_ and to assess the effect of Paeonin, flow cytometry experiments were performed with the FACSCalibur system (BD Biosciences, USA). For cell cycle analysis, the collected cells were washed with PBS, centrifuged and fixed in 70% ethanol overnight at −20 °C. Thereafter, cells were stained with 400 μl of staining solution containing 100 mg/ml of RNase A and 40 mg/ml of propidium iodide (PI) in the dark for 30 min at 37 °C, followed by flow cytometry analysis. For apoptosis analysis, the collected cells were similarly washed with PBS, centrifuged and resuspended in 250 μl of Annexin V binding buffer. After double staining with FITC-Annexin V and PI in the dark for 15 min at 37 °C using the FITC-Annexin V Apoptosis Detection Kit (Beyotime, China), according to the manufacturer’s protocols, cells were analyzed within 30 min on a FACScan flow cytometer. Additionally, the cell cycle-related proteins (e.g., Cyclin D1 and p27) and apoptosis-related proteins (e.g., Bcl2, Caspase3, Caspase8 and Bax) were also measured by WB, operated as mentioned above, to further reveal the distributions of cell cycle and apoptosis in GES-1 cells treated with H_2_O_2_ alone and H_2_O_2_ + Paeonin.

### Statistical analysis of the data

All data in this study were analyzed with SPSS 18.0 software (IBM SPSS, USA). All experiments were conducted, as at least three biological replicates, and the data presented as mean ± standard deviation (SD). Differences between more than two groups were assessed by one-way analysis of variance (ANOVA). Comparisons between two groups were evaluated using two-tailed Student t test. Significance was established at *P* value less than 0.05.

### Availability of data and materials

Please contact author for data requests.

## Results

### Oxidative damage model establishmentin GES-1 cells

**Figure 1:**
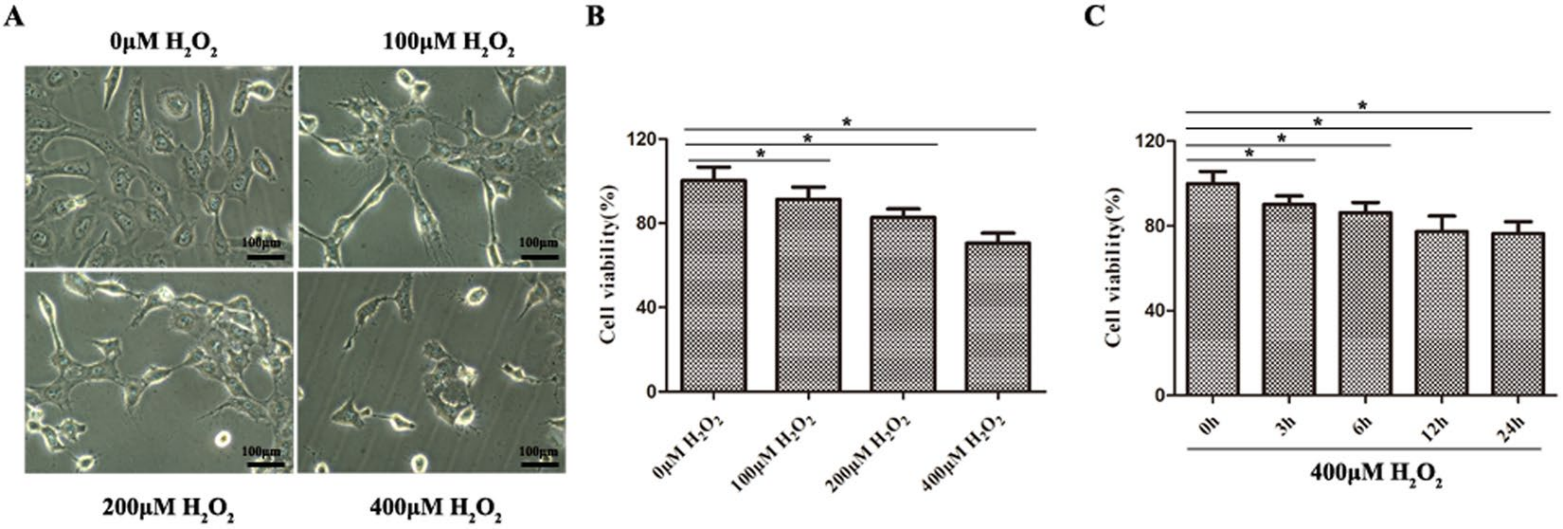
Oxidative damage model induced by H_2_O_2_was established in GES-1 cells. (**A**) Morphological changes in GES-1 cells exposed to 4 different concentrations of H_2_O_2_, including 0 μM, 100 μM, 200 μM and 400 μM. (**B**) The effects of various H_2_O_2_ concentrations on cell viability in GES-1 cells, as determined by MTT assay. The cell viability was gradually decreased in a dose-dependent manner; *P < 0.05. (**C**) The effects of 400 μM H_2_O_2_ on cell viability in GES-1 cells for 0 h, 3 h, 6 h, 12 h and 24 h. The cell viability gradually declined in a time-dependent manner; *P < 0.05.

To investigate the oxidative damage of H_2_O_2_, we treated GES-1 cells with H_2_O_2_ at different concentrations. It was found that GES-1 cells with 0 μM H_2_O_2_ treatment presented morphologically normal, while GES-1 cells with other concentrations of H_2_O_2_ treatment showed an incomplete cellular morphology, especially in the 400 μM H_2_O_2_ group (Fig. 1A). Moreover, the ratio of cell viability in the 400 μM H_2_O_2_ group was significantly lower than that in the other three groups (Fig. 1B). Additionally, it was also discovered that 400 μM H_2_O_2_ induced a decreasing ratio of cell viability in a time-dependent manner (Fig. 1C). Therefore, we chose 400 μM, as an optimal dose and 24 h, as an optimal period, for the subsequent experiments.

### Four extracted pigments from the potatoes repaired oxidative damage in GES-1 cells treated with H_2_O_2_

**Figure 2:**
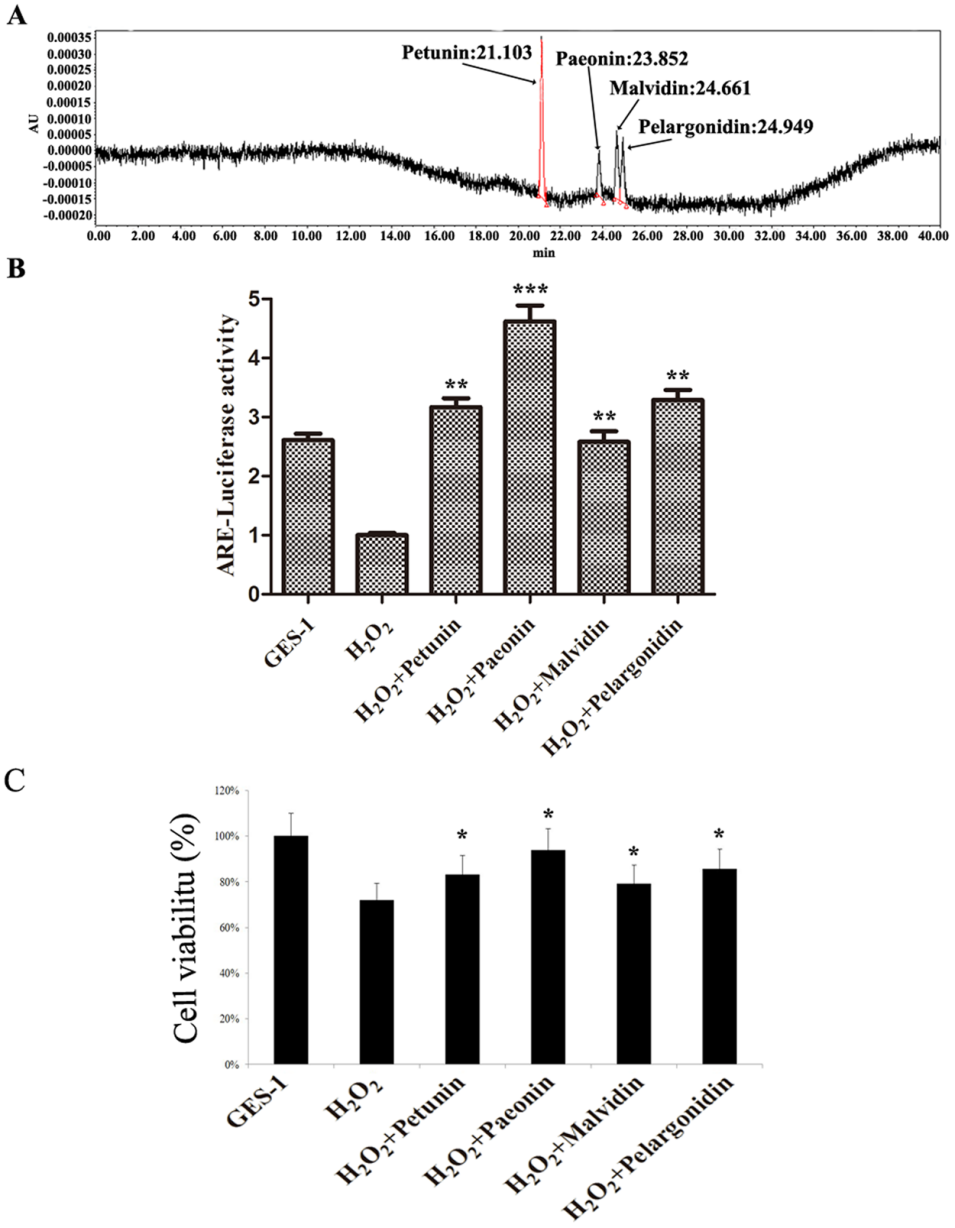
The functions of extracted pigments from potatoes in H_2_O_2_-treated GES-1 cells. (**A**) The pigments isolated from potatoes were detected by HPLC. There were four significant peaks (i.e., Petunin, Paeonin, Malvidin and Pelargonidin) between 20 min and 26 min. (**B**) ARE-luciferase activity was examined in H_2_O_2_-treated GES-1 cells pre-incubated with the four pigments extracted from potatoes. Compared to the GES-1 and H_2_O_2_ groups, ARE-luciferase activity was elevated by the four extracted pigments. The highest ARE-luciferase activity was induced by Paeonin in H_2_O_2_-treated GES-1 cells; *P < 0.05, **P < 0.01 or ***P < 0.001 versus the H_2_O_2_ group.

Our results revealed that four pigments, namely, Petunin, Paeonin, Malvidin and Pelargonidin, were isolated from potatoes (Fig. 2A). To determine whether these four pigments could alleviate oxidative damage in GES-1 cells with H_2_O_2_ treatment, GES-1 cells were pre-incubated with these four pigments. The results showed that compared to the GSE-1 and H_2_O_2_ groups, these four pigments, particularly Paeonin, remarkably promoted the ARE-luciferase activity. However, the ARE-luciferase activity represents an anti-oxidative status in cells; thereby, our results suggested that the four pigments could reduce H_2_O_2_-induced cellular oxidative stress injury. Meanwhile, Paeonin was selected as an optimal pigment for the subsequent experiments due to its activation of the highest signal of the ARE-luciferase reporter (Fig. 2B).

### The anti-oxidative and cell viability promotion effects of Paeonin

**Figure 3:**
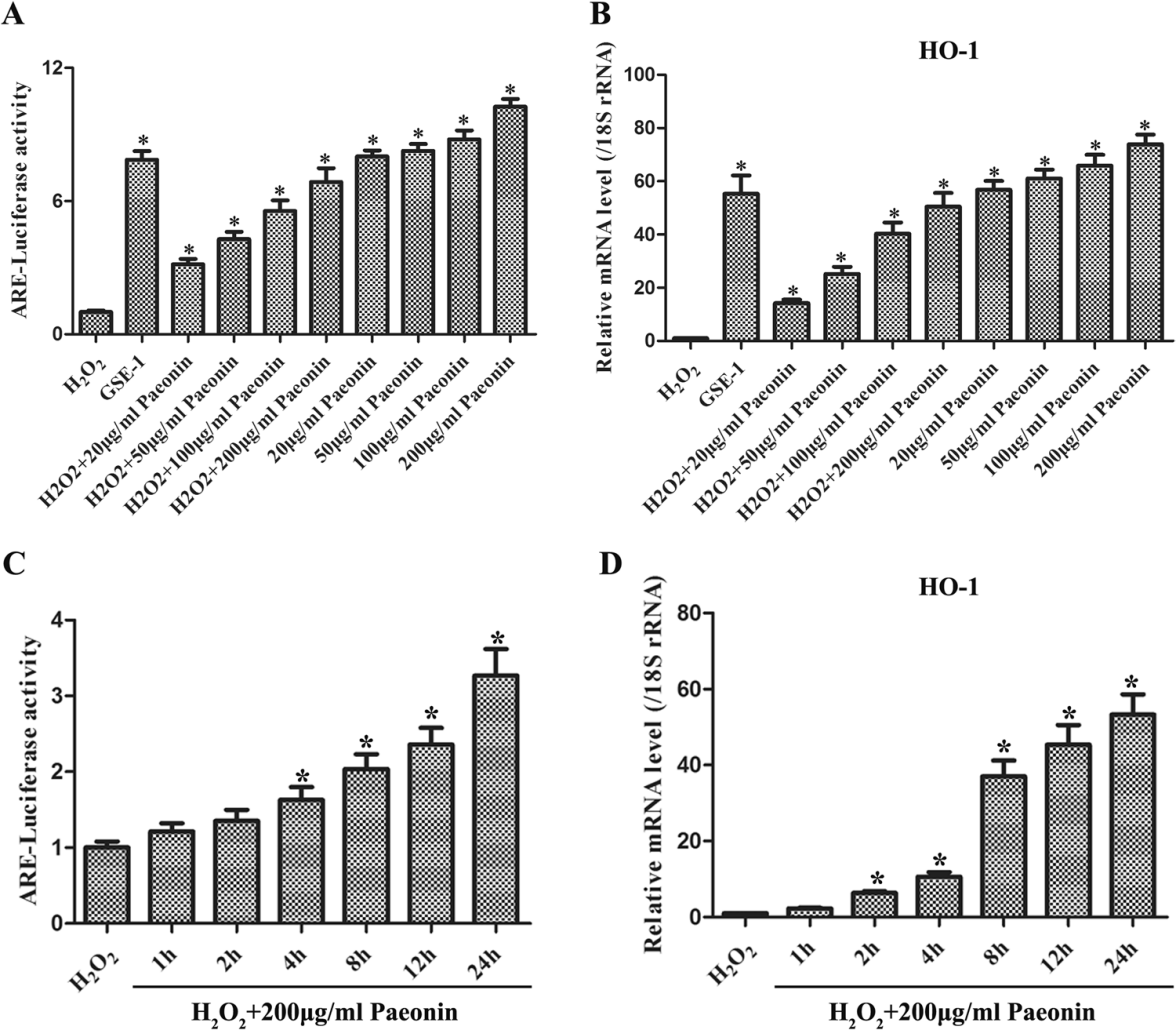
The anti-oxidative roles of Paeonin. (**A**) ARE-luciferase activity was measured by luciferase assay in H_2_O_2_-stimulated GES-1 cells with different Paeonin concentrations, including 20 μg/ml, 50 μg/ml, 100 μg/ml and 200 μg/ml. With the concentrations of Paeonin increased, the ARE-luciferase activity was also elevated. (**B**) HO-1 mRNA was determined by qRT-PCR in H_2_O_2_-incubated GES-1 cells with different Paeonin concentrations. HO-1 mRNA expressions and Paeonin concentrations had a positive correlation. (**C**) ARE-luciferase activity was detected by luciferase assay in H_2_O_2_-stimulated GES-1 cells with 200 μg/ml Paeonin for 1 h, 2 h, 4 h, 8 h, 12 h and 24 h. With the treatment time prolonged, the ARE-luciferase activity was also up-regulated. (**D**) HO-1 mRNA was tested by qRT-PCR in H_2_O_2_-incubated GES-1 cells with 200 μg/ml Paeonin for 1 h, 2 h, 4 h, 8 h, 12 h and 24 h. HO-1 mRNA expressions and the treatment time had a positive correlation; *P < 0.05 versus the H_2_O_2_ group.

**Figure 4:**
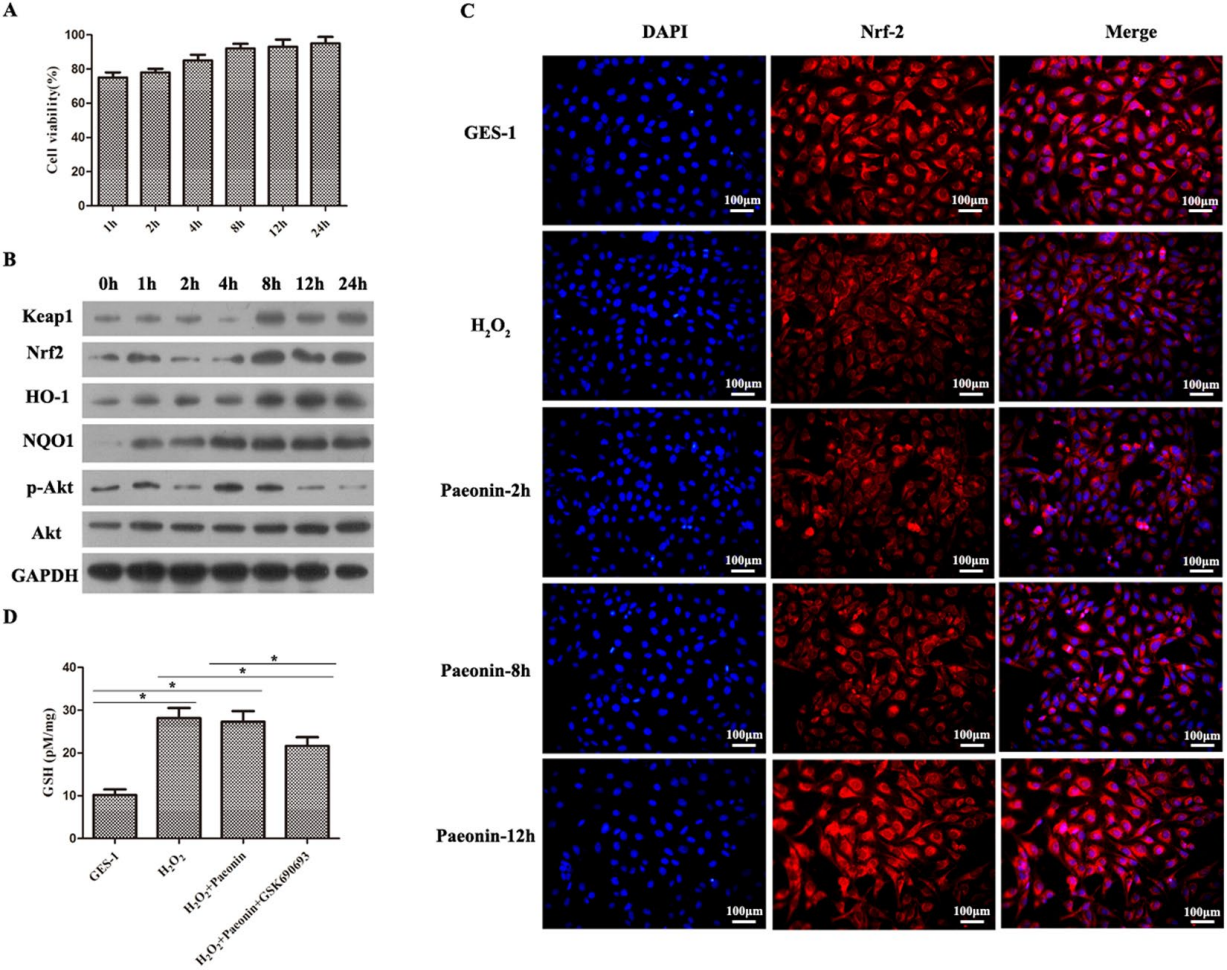
Paeonin exerted an anti-oxidative role by PI3K/Akt-mediated Nrf2 signaling pathway. (**A**) Cell viability was tested by MTT assay in H_2_O_2_-incubated GES-1 cells with 200 μg/ml Paeonin for 0 h, 1 h, 2 h, 4 h, 8 h, 12 h and 24 h. The cell viability presented a time-dependent increase. (**B**) PI3K/Akt-mediated Keap-1 Nrf2 signaling pathway-related protein expressions were measured by WB in H_2_O_2_-exposed GES-1 cells with 200 μg/ml Paeonin for 0 h, 1 h, 2 h, 4 h, 8 h, 12 h and 24 h. (**C**) The location expression of Nrf2 was examined by IF in H_2_O_2_-stimulated GES-1 cells with 200 μg/ml Paeonin for 2 h, 8 h and 12 h. The cell nucleus was stained into blue, while Nrf2 protein was stained into red. (**D**) GSH content was detected in the GSE-1, H_2_O_2_, H_2_O_2_ + Paeonin and H_2_O_2_ + Paeonin + GSK690693 groups. GSH content in the three experiment groups was higher than that in the GSE-1 group; *P < 0.05.

To confirm the anti-oxidative effect of Paeonin, we adopted different Paeonin concentrations to pre-treat GES-1 cells for different times. Our data showed that the ARE-luciferase activity (Fig. 3A) and HO-1 mRNA expression (Fig. 3B) in GES-1 cells, pre-incubated with different concentrations of Paeonin before treatment with 400 μM H_2_O_2_, were both gradually increased in a dose-dependent manner. Next, we chose 200 μg/ml Paeonin for pre-treatment in GES-1 cells and then used 400 μM H_2_O_2_ to incubate for 0 h, 1 h, 2 h, 4 h, 8 h, 12 h and 24 h. It was shown that the ARE-luciferase activity (Fig. 3C) and HO-1 mRNA expression (Fig. 3D) were also notably elevated with time in GES-1 cells with H_2_O_2_ + Paeonin treatment. Additionally, the ratio of cell viability was markedly up-regulated over time in H_2_O_2_ + Paeonin-treated GES-1 cells (Fig. 4A). Hence, these findings indicated that Paeonin not only exerted an anti-oxidative role but could also promote cellular survival in oxidative damage cell model.

### Nrf2 signaling pathway and PI3K/Akt signaling pathway might be involved in the anti-oxidative mechanisms of Paeonin

To further explore whether Paeonin activated the Nrf2 signaling pathway to improve its anti-oxidative effects, we first examined the expression levels of Nrf2 signaling-related proteins by WB. Our results uncovered that Keap1, Nrf2, HO-1 and NQO1 protein expressions presented weaker expression in 0 h, 1 h, 2 h and 4 h, but these protein expressions were obviously augmented in 8 h, 12 h and 24 h (Fig. 4B). Nevertheless, p-Akt protein expression was gradually increased from 0 h to 4 h and gradually decreased from 4 h to 24 h, while there were no significant alterations in Akt protein expression at all time points (Fig. 4B). Moreover, the images of IF also showed that the fluorescence intensity of Nrf2 was dramatically up-regulated over time (Fig. 4C). Thus, these results implied that Paeonin activated the Nrf2 signaling pathway with time. Subsequently, we used an inhibitor of PI3K/Akt signaling pathway (i.e., GSK690693) to demonstrate the proliferation promotion effects of Paeonin. Our data revealed that in comparison with the H_2_O_2_ and H_2_O_2_ + Paeonin groups, treatment with H_2_O_2_ + Paeonin + GSK690693 resulted in a relative decline in intracellular GSH content (Fig. 4D). Therefore, the data concluded that PI3K/Akt signaling pathway might be implicated in oxidative damage repair in H_2_O_2_-treated GSE-1 cells.

### Paeonin treatment enhanced cell proliferation and suppressed cell apoptosis

**Figure 5:**
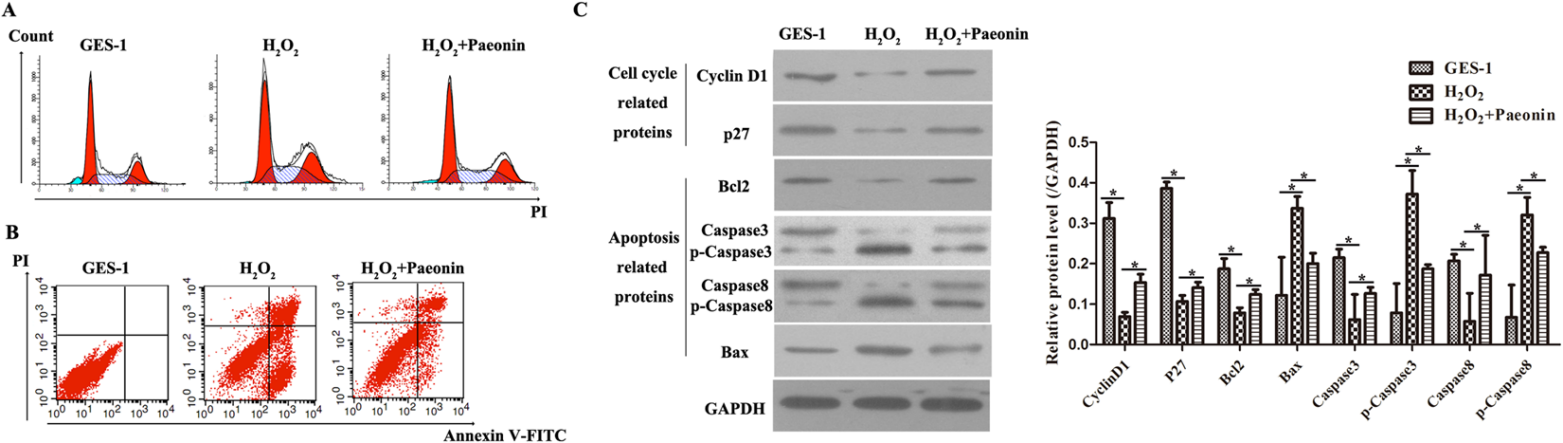
The impact of Paeonin in cell cycle and apoptosis. GSE-1 cells were divided into three groups: GSE-1 group, H_2_O_2_ group and H_2_O_2_ plus 200 μg/ml Paeonin group. (**A**) Cell cycle was examined by flow cytometry. The representative images of cell cycle are presented. (**B**) Apoptosis was measured by flow cytometry. The representative images of apoptosis are exhibited. (**C**) Cell cycle and apoptosis- related proteins were detected by WB. Among these, cyclin D1 and p27 were cell cycle-related proteins, while Bcl2, Caspase3, p-Caspase3, Caspase8, p-Caspase8 and Bax were apoptosis-related proteins. GAPDH was considered the endogenous reference. (**D**) The relative expressions of cyclin D1, p27, Bcl2, Caspase3, p-Caspase3, Caspase8, p-Caspase8 and Bax proteins were calculated and presented as a histogram.

To further elaborate the repair mechanism of Paeonin in H_2_O_2_-treated GSE-1 cells, cell cycle and apoptosis were measured by flow cytometry. As illustrated in Fig. 5A, H_2_O_2_ remarkably caused cell cycle arrest; but when pre-treated with Paeonin, cell cycle was improved. Additionally, it was also found that H_2_O_2_ treatment markedly promoted cell apoptosis in GES-1 cells, while cell apoptosis was down-regulated in H_2_O_2_ + Paeonin-treated GSE-1 cells (Fig. 5B). Furthermore, the protein expression levels of Cyclin D1, p27, Bcl2, total Caspase3 and total Caspase8 in the H_2_O_2_ + Paeonin group were notably higher than those in the H_2_O_2_ group; while the protein expression level of Bax, p-Caspase3 and p-Caspase8 in the H_2_O_2_ + Paeonin group was markedly lower than that in the H_2_O_2_ group (Fig. 5C). The relative protein expression levels quantified by densitometry using Image J Analysis software were presented in Fig. 5D. Thus, these results suggested that Paeonin could accelerate cell proliferation and decelerate apoptosis in H_2_O_2_-treated GSE-1 cells.

## Discussion

Oxidative stress plays a role in the pathogenesis of gastric diseases. In this study, our results firstly showed that GES-1 cells significantly presented a morphological change and a dose- and time-dependent cell viability loss after H_2_O_2_ exposure, which is in agreement with other studies^19,20^. Thereby, these results implied that a model of oxidative stress in GES-1 cells was successfully established. Then, pigments were extracted from potatoes and then purified by HPLC. Moreover, it was found that among four isolated pigments, Paeonin induced the highest anti-oxidant effects on H_2_O_2_-exposed GES-1 cells. Presently, it is widely recognized that increased dietary anti-oxidant intake is an optimal choice to reduce the risk of gastric mucosal oxidative injury^16,17,21^. Moreover, in our previous study, it has been demonstrated that anthocyanins extracted from ‘Jianchuanhong’ and ‘Zhuanxinwu’ potatoes had strong antioxidant activity in a concentration dependent manner and revealed a significant free radical scavenging capacity 10.7~31.3 times higher than that of vitamin C^22^, which indicated that the potatoes possessed an anti-oxidant effects. Therefore, to further confirm the role of Paeonin in protecting GES-1 cells against oxidative injury, ARE-luciferase activity and HO-1 mRNA expression, which represented anti-oxidant ability in cells, were examined by luciferase assay and qRT-PCR, respectively, in H_2_O_2_-treated GES-1 cells. Our data revealed that Paeonin up-regulated the ARE-luciferase activity and HO-1 mRNA expression in H_2_O_2_-treated GES-1 cells in a dose- and time-dependent manner, suggesting that Paeonin might exert an anti-oxidative role in H_2_O_2_-incubated GES-1 cells. Furthermore, it was also discovered that cell survival rates were gradually elevated with Paeonin-treated time in H_2_O_2_-incubated GES-1 cells, further indicating that Paeonin alleviated the cell damage in GES-1 cells with H_2_O_2_ treatment.

To further excavate the potential mechanism of Paeonin in resisting oxidative damages, increasing interest was mainly focused on Nrf2 signaling pathway, which is a redox-sensitive transcriptional factor that localizes to the cytoplasm and interacts with Keap1under normal conditions^23^. Once activated, Nrf2 translocates into the nucleus, binds the antioxidant response element of the target gene promoter that leads to transcription of various antioxidant and detoxifying enzymes, and ultimately exhibits cytoprotective effects. Accumulating studies have shown that activation of the Nrf2 pathway is therefore a therapeutic target for treatment of gastric diseases^24^ In this work., it was uncovered that Keap-1, Nrf2, HO-1 and NQO1 were obviously elevated in H_2_O_2_-stimulated GES-1 cells with Paeonin-treated time. Furthermore, the fluorescence intensity of Nrf2 in H_2_O_2_-treated GES-1 cells was also augmented with Paeonin-treated time. Nrf2, identified as a transcription factor, dissociates from Kelch-like ECH-associated protein-1 (Keap1) under oxidative stress conditions and then translocates into the nucleus, where it binds to the ARE, resulting in the transcriptional activation of anti-oxidative genes, including HO-1 and NQO1^25,26^. Furthermore, over the past decades, various researchers have indicated that enhancement of Nrf2 in nucleus or improvement of its down-stream genes effectively protects cells from cell damage induced by oxidative stress^25,27^. For instance, salidroside suppressed human umbilical vein endothelial cell (HUVEC) injury induced by oxidative stress through activating the Nrf2 signaling pathway^28^, and chlorogenic acid protected against H_2_O_2_-induced oxidative stress in mouse osteoblastic cell line MC3T3-E1 via PI3K/Akt-mediated Nrf2/HO-1 signaling pathway^29^. Thus, our results suggested that Paeonin might trigger Nrf2 signaling pathway to prevent oxidative injuries in H_2_O_2_-stimulated GES-1 cells. Additionally, activation of PI3K/Akt signaling is regarded as a crucial upstream event of activation of Nrf2 signaling pathway; thereby, PI3K/Akt signaling regulated anti-oxidative gene expressions in an Nrf2-dependent transcription fashion, in diverse cells, in response to oxidative stress^15,30^. Meanwhile, in the current study, p-Akt protein expression exhibited a parabolic curve trend with the peak at 8 h with Paeonin treatment in H_2_O_2_-exposed GES-1 cells. Additionally, compared to the H_2_O_2_ group and the H_2_O_2_ + Paeonin group, GSK690693, a PI3K/Akt signaling inhibitor, apparently suppressed the GSH content in the H_2_O_2_ + Paeonin + GSK690693 group. Nevertheless, accumulation of GSH levels in cells reflected inhibition condition of oxidative stress. Hence, these findings concluded that the anti-oxidative protective effects of Paeonin might be induced by PI3K/Akt-mediated Nrf2 signaling pathway.

## Conclusions

Eventually, evaluations of cell cycle and apoptosis were detected by flow cytometry and WB. Our data demonstrated that Paeonin treatment could remarkably ameliorate the impacts of H_2_O_2_ in cell cycle arrest and apoptosis of GES-1 cells. Currently, it has been verified that H_2_O_2_-induced cell cycle arrest and apoptosis are considered key indexes of cell damages^30–32^. Moreover, compared to H_2_O_2_-stimulated GES-1 cells, WB experiments also validated that Paeonin promoted cell cycle-related Cyclin D1 and p27 and anti-apoptotic-related Bcl2 protein expressions and inhibited pro-apoptotic-related Bax, p-Caspase3 and p-Caspase8 protein expression. Therefore, these results implied that Paeonin was effective against H_2_O_2_-induced cell damage.

In summary, we provided the first evidence that Paeonin extracted from potatoes elicited a cytoprotective effect against H_2_O_2_-induced oxidative stress damages through a mechanism that depended, at least in part, on the activation of the PI3K/Akt-mediated Nrf2 signaling pathway. This study highlighted the value of Paeonin, as a new and promising target for reversing oxidative stress in gastric diseases^33^.
